# A Curious Case of Multimorbidity in a Patient With Goldenhar Syndrome Presenting With Vomiting

**DOI:** 10.7759/cureus.72662

**Published:** 2024-10-29

**Authors:** Rahul Borra, Alisher Hamidullah, Tong Ren, Venkat Bhaskara

**Affiliations:** 1 Internal Medicine, HCA Healthcare, University of South Florida Morsani College of Medicine GME: Oak Hill Hospital, Brooksville, USA; 2 Internal Medicine, Dr. Kiran C. Patel College of Osteopathic Medicine, Nova Southeastern University, Fort Lauderdale, USA

**Keywords:** anatomical variability, gallstone pancreatitis, goldenhar syndrome surgery, hepatitis c (hcv) infection, primary biliary cirrhosis (pbc)

## Abstract

Goldenhar syndrome, also known as oculo-auriculo-vertebral dysplasia or hemifacial microsomia, is a rare congenital anomaly involving the first and second branchial arches. In this case report, we present a distinctive instance of a 43-year-old male with Goldenhar syndrome who presented with nausea and recurrent bilious vomiting. Initial diagnostic imaging raised concerns about pancreatitis, leading to a comprehensive evaluation that revealed gallstone pancreatitis as the cause of his symptoms. Despite the seemingly straightforward diagnosis, the case was complicated by anatomical abnormalities that required multiple interventions and led to additional complications related to the patient’s underlying condition. The complexities of this case highlight the potential challenges in managing what may initially appear to be uncomplicated presentations in patients with Goldenhar syndrome, emphasizing the critical importance of a multidisciplinary approach. This report underscores the need for timely, well-reasoned clinical strategies to ensure optimal care and prevent adverse outcomes in such patients.

## Introduction

Maurice Goldenhar, a distinguished Belgian ophthalmologist and general practitioner, first described Goldenhar syndrome in 1952 after observing pediatric cases of hemifacial microsomia accompanied by oral, aural, and mandibular developmental anomalies. Although the exact etiology remains unclear, the prevailing theory suggests that the syndrome arises from an imbalance in cell distribution during the blastogenesis phase of embryonic development [1]. This relatively uncommon congenital anomaly typically presents with unilateral abnormalities in structures derived from the first and second branchial arches, affecting the eyes, ears, soft palate, and jaw. In some cases, additional systems - including the vertebral, cardiac, gastrointestinal, and central nervous systems - may also be involved. The syndrome is often characterized by a classical triad of epibulbar dermoids, accessory auricular appendages, and aural fistulas [2].

Due to limited comprehensive data, prevalence estimates of Goldenhar syndrome vary widely, ranging from 1:3,000 to 1:5,000 [3], although some sources suggest rarer estimates between 1:35,000 and 1:56,000. The condition shows a slight male predominance, with a male-to-female ratio of 3:2.

In this report, we describe the unique presentation and hospital course of a 43-year-old male with Goldenhar syndrome who initially presented with symptoms suggestive of gallstone pancreatitis but was later found to have hepatitis of unknown etiology.

## Case presentation

The patient, a 43-year-old man with a complex medical history including Goldenhar syndrome, type 2 diabetes mellitus, a repaired “hole in the heart,” scoliosis, prior lung surgery, and mutism, presented to the emergency room with intractable nausea and vomiting. Due to his mutism, the medical history was obtained from his mother, who reported sudden retching followed by multiple episodes of bilious vomiting a few hours prior to the emergency department visit. The patient’s mother denied any alcohol or drug use, as well as any sick contacts. The patient had no reported fever, fatigue, cough, abdominal pain, weight loss, or illicit drug use.

Upon physical examination, the patient exhibited a temperature of 36.4 °C, a pulse of 94 beats per minute, a respiratory rate of 18 breaths per minute, a blood pressure of 128/79 mmHg, and an oxygen saturation of 95% on room air. Although alert, the patient was in mild distress with active retching. He had low-set eyes bilaterally, was normocephalic, and displayed no scleral icterus. His ears were low-set and small, and no thyromegaly was observed. A holosystolic murmur with an S1 upstroke was detected. Lung auscultation revealed clear breath sounds bilaterally, without wheezing or rhonchi. The abdominal examination showed scars and distension, with normoactive bowel sounds throughout all four quadrants and a non-tender abdomen upon palpation. No edema was observed in the upper and lower extremities.

The patient’s laboratory findings revealed leukocytosis, a normal hemoglobin level, and a normal platelet count (Table 1). The chemistry panel indicated an elevated blood glucose level (Table 2). The liver panel exhibited total bilirubin within normal limits, but elevated liver function tests for aspartate aminotransferase (AST), alanine transaminase (ALT), and alkaline phosphatase (ALP; Table 2). Notably, amylase and lipase levels were severely elevated, suggesting pancreatic involvement. Finally, lactic acid levels were found to be slightly elevated as well (Table 2).

**Table 1:** Complete blood count The complete blood count was performed initially to establish the patient’s baseline laboratory values prior to investigating the potential cause of his nausea and vomiting. The results indicated a leukocytosis of 15.7, which may suggest an infectious etiology for his vomiting.

Lab values	Patient’s lab value	Reference value
White blood cell	15.7 × 10^9^/L	4-11 × 10^9^/L
Red blood cell	5.47 × 10^9^/L	4.63-6.08 × 10^9^/L
Hemoglobin	14.8 g/dL	13.7-17.5 g/dL
Hematocrit	47.30%	40-51%
Mean corpuscular volume	86.5 fL	79-92.2 fL
Mean corpuscular hemoglobin	27.1 pg	25.7-32.2 pg
Mean corpuscular hemoglobin concentration	31.3 g/dL	32.3-36.5 g/dL
Red cell distribution width	13.50%	11.6-14.4%
Platelet count	192 × 10^9^/L	150-140 × 10^9^/L
Mean platelet volume	11.1 fL	9.4-12.4 fL

**Table 2:** Complete metabolic panel The patient’s complete metabolic panel showed elevated amylase and lipase levels, suggesting that the cause of the vomiting may be related to pancreatic dysfunction. ALT, alanine transaminase; AST, aspartate aminotransferase; BUN, blood urea nitrogen; Cr, creatinine; GFR, glomerular filtration rate

Lab values	Patient’s lab value	Reference value
Sodium	138 mmol/L	136-145 mmol/L
Potassium	3.9 mmol/L	3.5-5.1 mmol/L
Chloride	103 mmol/L	98-107 mmol/L
Bicarbonate	29 mmol/L	21-32 mmol/L
Anion gap	6 mmol/L	7-16 mmol/L
BUN	14 mg/dL	7-18 mg/dL
Creatinine	1.1 mg/dL	0.6 mg/dL
Estimated GFR	85.4 mL/min	>90.0 mL/min
Bun/Cr ratio	12 mg/dL	4-33 mg/dL
Glucose	176 mg/dL	74-106 mg/dL
Serum osmolality	322 mOsm/kg	276-300 mOsm/kg
Calcium	8.9 mg/dL	8.5-10.1 mg/dL
Total bilirubin	0.5 mg/dL	0.2-1 mg/dL
AST	45 units/L	15-37 units/L
ALT	59 units/L	13-61 units/L
Alkaline phosphatase	130 units/L	45-117 units/L
Total protein	8.4 g/dL	6.4-8.2 g/dL
Albumin	3.8 g/dL	3.4-5 g/dL
Globulin	4.6 g/dL	1.4-4.8 g/dL
Amylase	1,942 units/L	25-115 units/L
Lipase	2,691 units/L	13-75 units/L
Lactic acid	3 mmol/L	0.4-2 mmol/L

**Figure 1:**
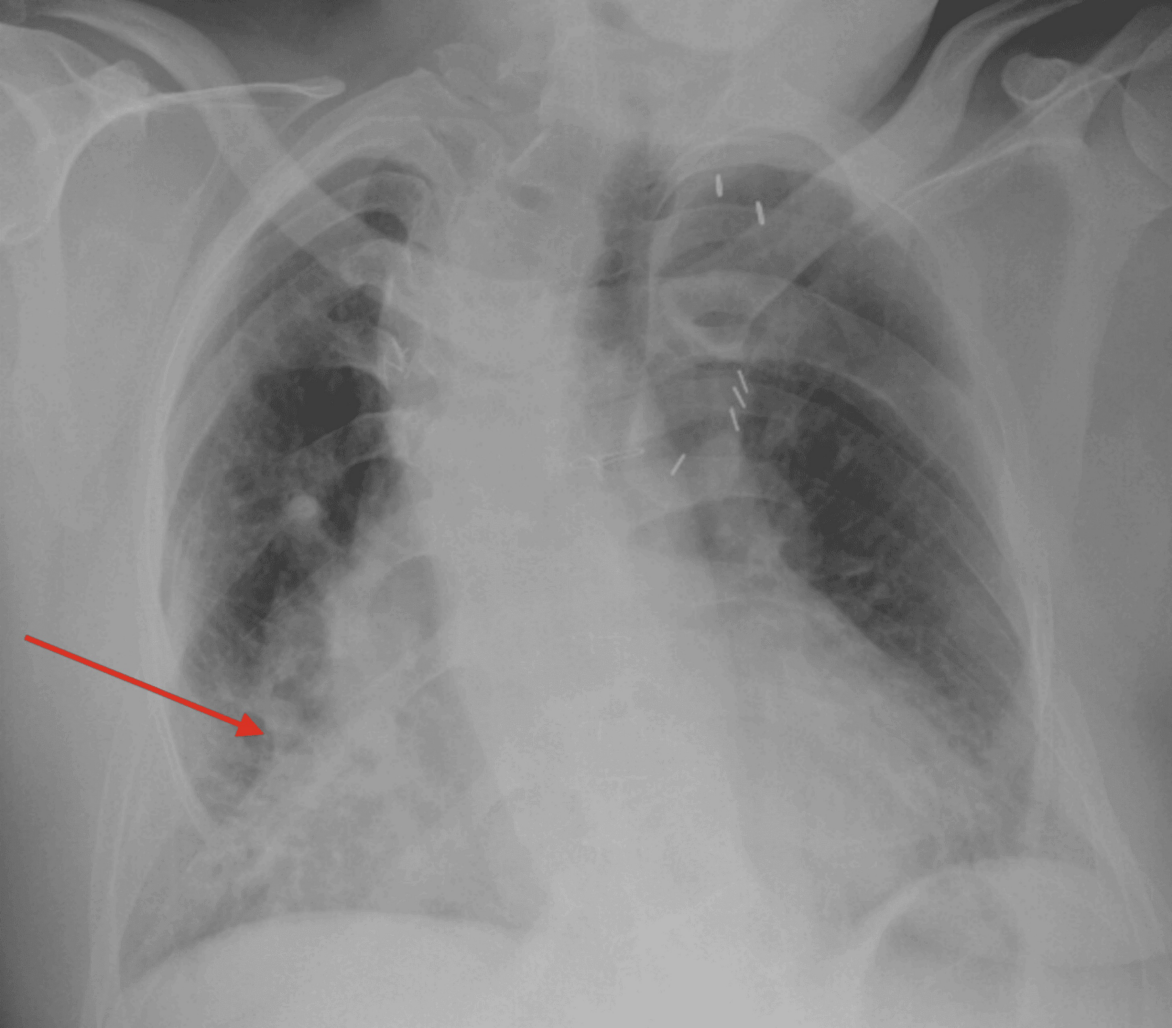
Initial X-ray demonstrating opacification of the right middle and right lower lobes

**Figure 2:**
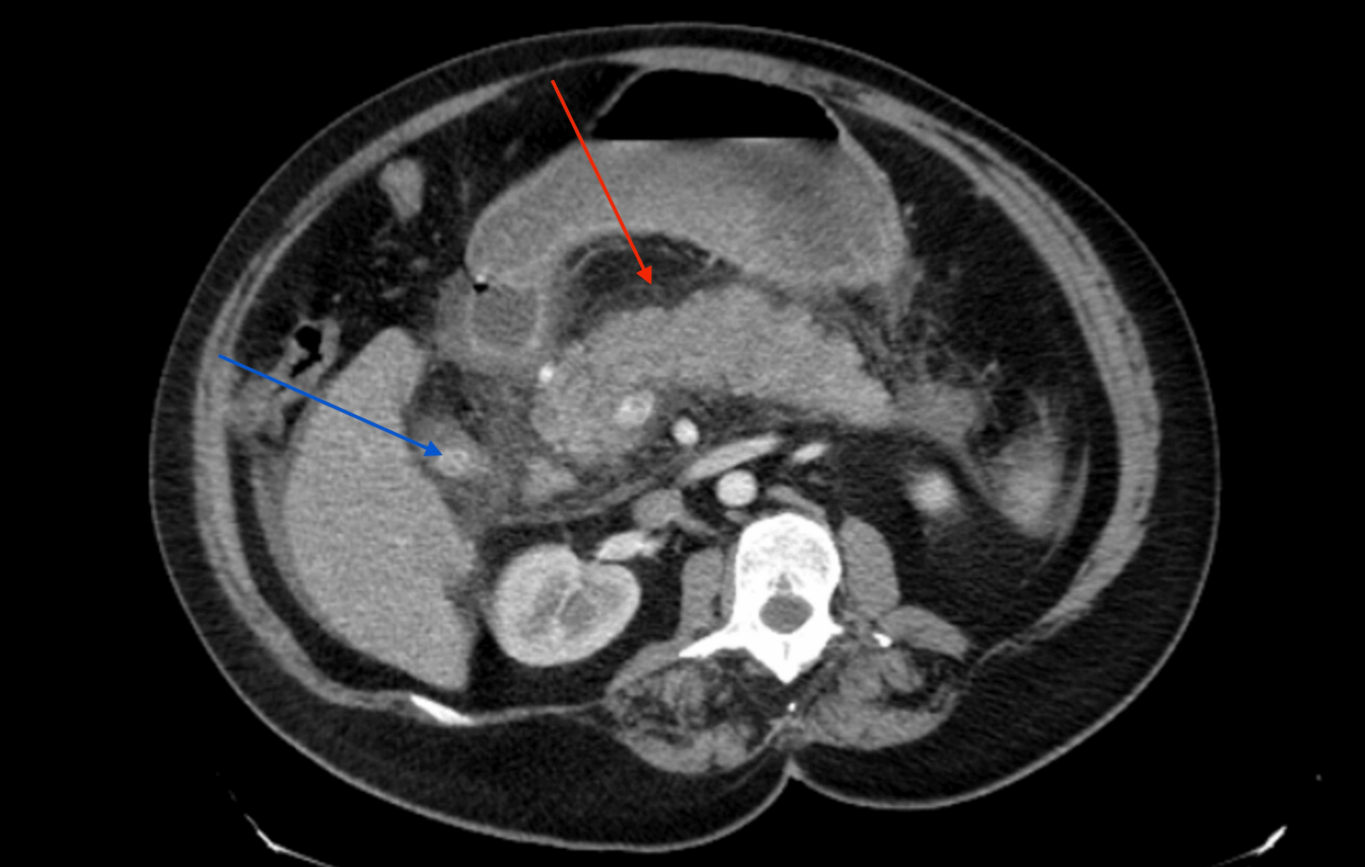
CT scan of the abdomen and pelvis The red arrow indicates acute pancreatitis with peri-pancreatic edema and linear fluid, and the blue arrow indicates a 1.3 cm gallstone.

Radiological investigations revealed a chest X-ray showing dense opacification in the right middle and lower lobes (Figure 1). A chest CT scan indicated mild cardiomegaly with central pulmonary artery dilation. Abdominal CT findings demonstrated moderate acute pancreatitis with peri-pancreatic edema and linear fluid, accompanied by cholelithiasis (Figure 2). A right upper quadrant ultrasound showed an enlarged, heterogeneous pancreas consistent with pancreatitis, and a contracted gallbladder containing a 1.2 cm gallstone, with no evidence of biliary duct dilation.

These findings collectively illustrated a complex clinical scenario involving acute pancreatitis, cholelithiasis, and pulmonary complications in a patient with a history of Goldenhar syndrome and other comorbidities. The elevated liver enzymes, along with the abnormalities in pancreatic enzymes, further complicated the case, necessitating a comprehensive and multidisciplinary approach to diagnosis and management.

Clinical course and intervention

The initial treatment plan involved consulting surgery for a minimally invasive robotic-assisted cholecystectomy to address the suspected gallstone pancreatitis. However, post-laparoscopic cholecystectomy, the patient developed refractory hypotension despite fluid resuscitation. Further evaluation revealed intra-abdominal bleeding, with a significant drop in hemoglobin from 14.8 to 7.2.

**Figure 3:**
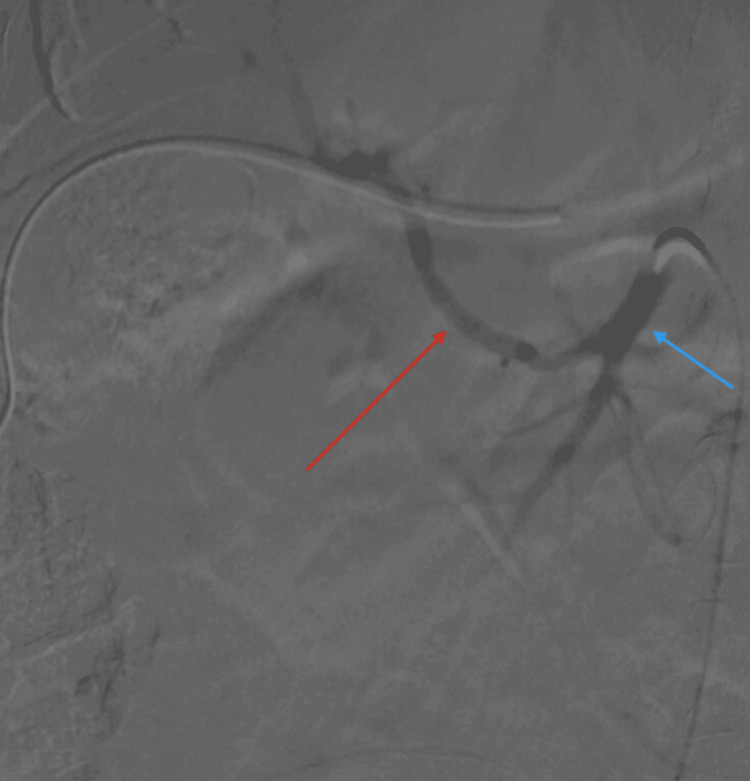
Angiogram of the mesenteric vessels The blue arrow indicates the superior mesenteric artery, and the red arrow indicates the common hepatic artery.

**Figure 4:**
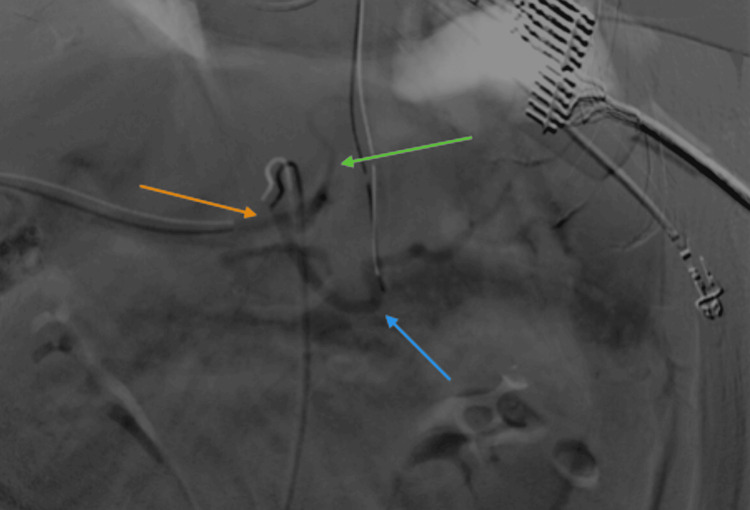
Angiogram of the mesenteric vessels The orange arrow indicates the celiac trunk, the blue arrow indicates the splenic artery, and the green arrow indicates the left gastric artery.

Given the urgency of the situation, a stat exploratory laparotomy was performed, which revealed no active bleeding but a substantial hematoma of 1250 ml within the abdominal cavity. Following the evacuation of the hematoma, the patient’s persistent hypotension prompted the scheduling of a mesenteric angiogram to identify the source of the bleeding. The angiogram revealed an anatomical variation, notably the common hepatic artery originating from the superior mesenteric artery (Figure 3, Figure 4), but no active bleeding source was identified.

The angiogram results highlighted the challenging anatomical variation, which potentially contributed to the complexity of the intra-abdominal bleeding. Although no active bleeding source was identified, the patient’s clinical course underscored the necessity for vigilant monitoring and consideration of alternative interventions in managing complications post-cholecystectomy.

**Figure 5:**
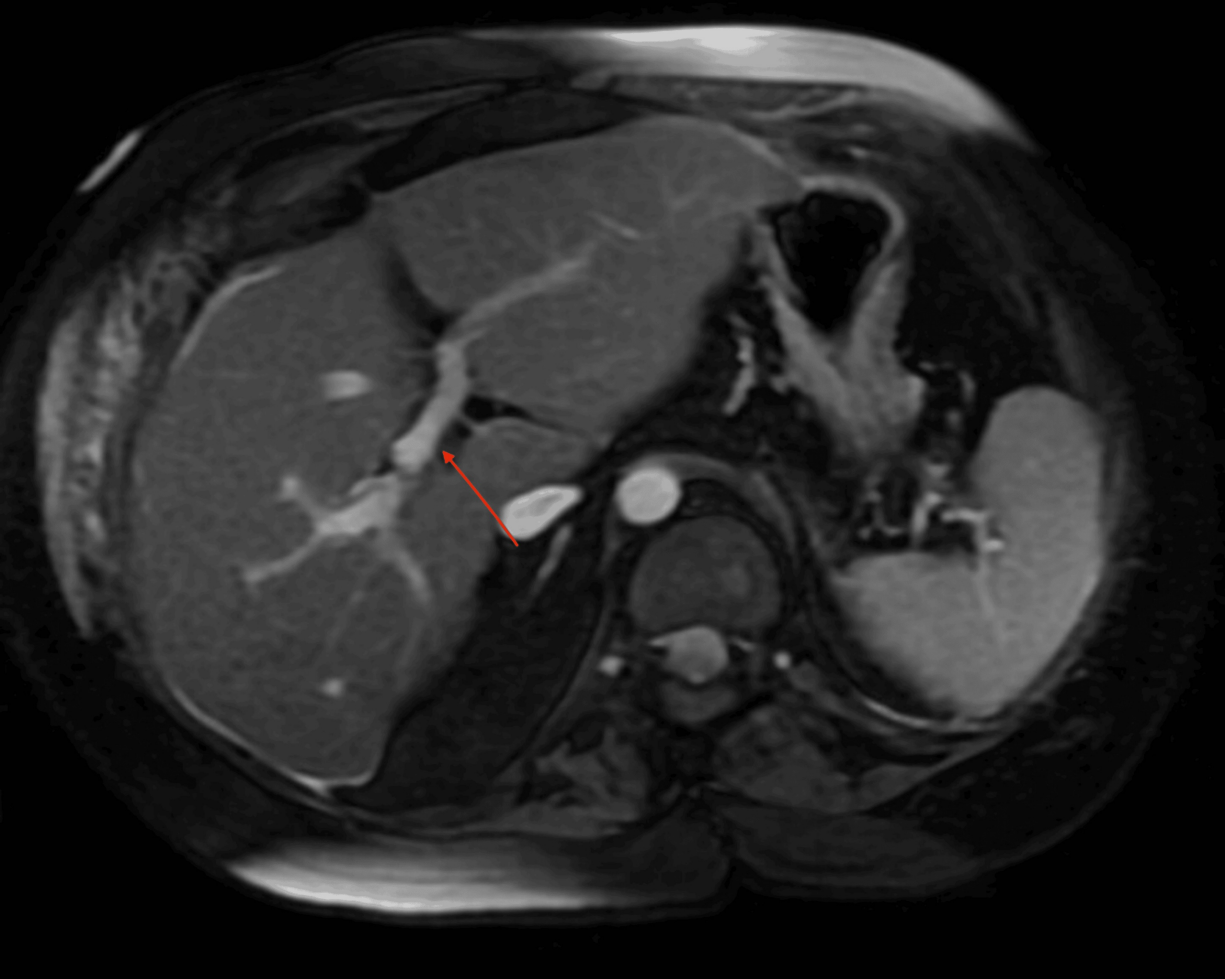
MRCP The red arrow indicates the non-obstructed portal vein. MRCP was performed due to the patient’s elevated LFTs. During the evaluation of the increased LFTs, imaging was conducted to rule out portal vein thrombosis and biliary duct obstruction as potential underlying causes. LFT, liver function test; MRCP, magnetic resonance cholangiopancreatography

Despite the cholecystectomy, the patient’s liver function tests displayed a concerning upward trend, further complicating the clinical course. The escalation in liver enzyme levels peaked at AST 859, ALT 749, ALP 297, and total bilirubin 2.1 mg/dL, persisting even after the discontinuation of hepatotoxic medications. The complexity of the case was further elucidated by the identification of hepatitis C, contributing to the ongoing hepatic challenges. The serological assessment was positive for anti-mitochondrial antibodies, while antinuclear antibodies (ANA) and anti-smooth muscle antibodies remained within the normal range. Imaging studies, including magnetic resonance cholangiopancreatography (Figure 5) and Doppler ultrasound, ruled out biliary ductal obstruction and portal vein thrombosis.

**Figure 6:**
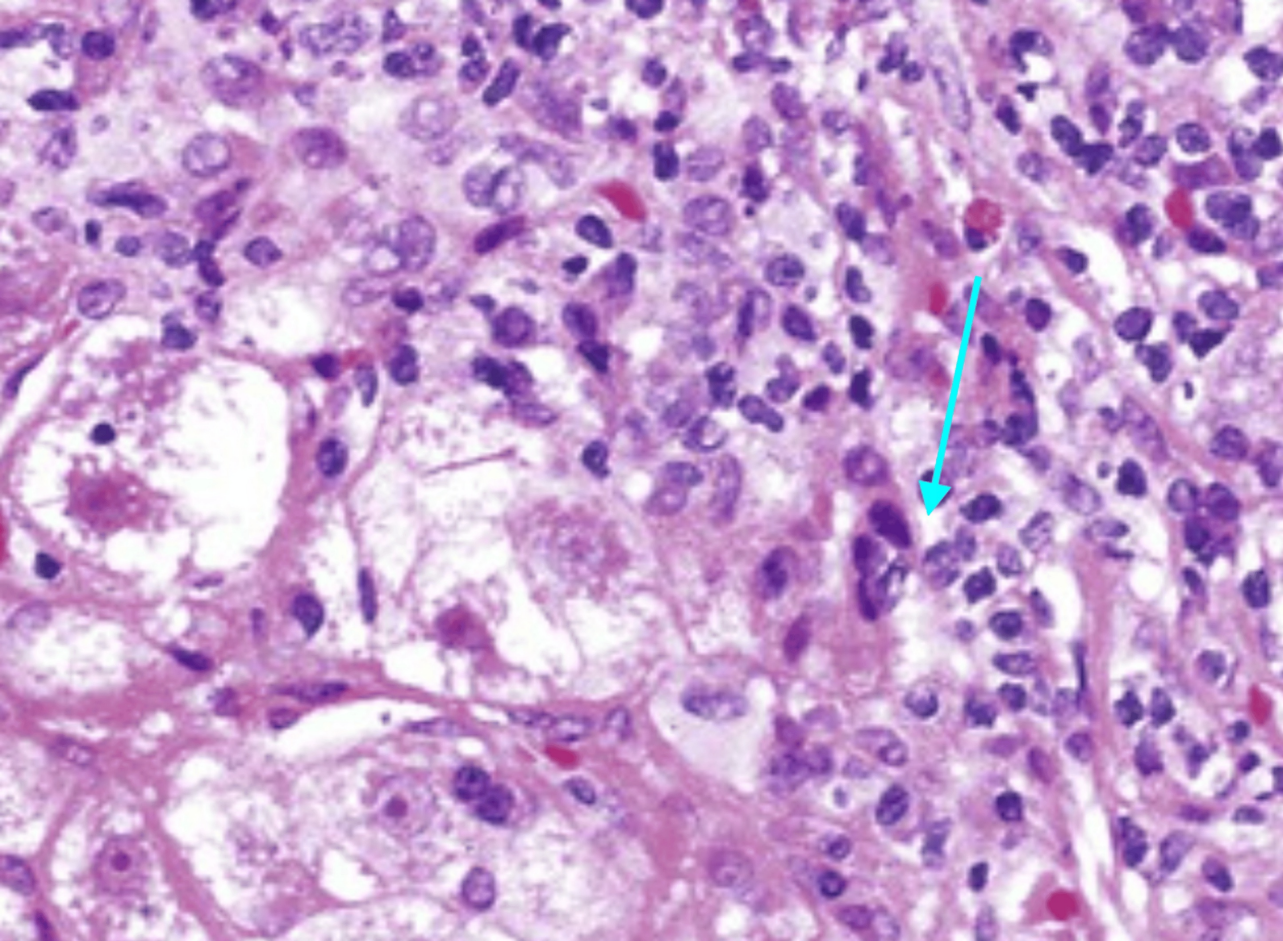
Liver biopsy showing mild lobular inflammation and scattered necrotic hepatocytes

A liver biopsy provided valuable insights, revealing mild lobular inflammation with scattered necrotic hepatocytes consistent with known viral hepatitis C (Figure 6). The histopathological assessment, graded as Grade 2 Stage II according to the Batts-Ludwig system, indicated no significant cholestasis or steatosis. Importantly, there was a notable absence of increased iron or intra-cytoplasmic alpha-1 antitrypsin globules. The viral load was quantified at over 3.4 million IU/mL.

This intricate clinical scenario underscores the complexity of managing liver dysfunction in the context of concurrent hepatitis C, primary biliary cirrhosis, and anatomical variation. The patient was discharged home with plans for follow-up with a gastroenterologist within the next one to two weeks. This post-discharge care was essential for monitoring the patient’s ongoing liver function and addressing any potential complications related to hepatitis C and post-cholecystectomy management.

## Discussion

We presented the case of a 43-year-old male with Goldenhar syndrome, a history of multiple comorbidities, and a complex clinical course involving gallstone pancreatitis, intra-abdominal bleeding post-cholecystectomy, and underlying hepatitis C, all of which posed significant diagnostic and management challenges. Several key aspects warrant discussion:

The patient’s underlying Goldenhar syndrome, characterized by craniofacial and vertebral anomalies [4,5], adds complexity to the case. The association of Goldenhar syndrome with other conditions, such as scoliosis and congenital heart defects [6,7], underscores the importance of a comprehensive understanding of the patient's medical history in guiding clinical decisions.

The initial presentation with symptoms suggestive of gallstone pancreatitis and subsequent intra-abdominal bleeding post-cholecystectomy highlights the intricacies of managing patients with congenital anomalies. The anatomical variations observed during surgery and the findings from the subsequent angiogram underscore the need for adaptability in surgical interventions and vigilant postoperative monitoring.

The discovery of underlying hepatitis C significantly complicates the patient’s clinical course. The persistence of elevated liver enzymes following cholecystectomy, despite the discontinuation of hepatotoxic medications, necessitates careful evaluation of hepatic involvement. The liver biopsy findings provide crucial insights into chronic hepatitis C, informing further therapeutic considerations.

Serological assessments, including the presence of anti-mitochondrial antibodies and the absence of ANA and anti-smooth muscle antibodies, contribute to characterizing the underlying autoimmune and viral components. The liver biopsy results indicate Grade 2 Stage II chronic hepatitis C (Batts Ludwig system) [8], without significant cholestasis or steatosis, further enhancing the understanding of the patient’s hepatic condition.

The patient’s discharge with a planned follow-up with a gastroenterologist within one to two weeks emphasizes the importance of ongoing monitoring and management. A multidisciplinary approach involving gastroenterology, surgery, and potentially hepatology is crucial for addressing the diverse aspects of the patient’s health challenges.

This case underscores the significance of patient-centered care, considering the unique medical history, congenital anomalies, and the complex interplay of conditions. It emphasizes the need for a collaborative, patient-specific approach to ensure optimal outcomes in patients with congenital anomalies.

## Conclusions

Our case exemplifies the intricate nature of managing a patient with Goldenhar syndrome and multiple comorbidities, including gallstone pancreatitis and hepatitis C. As a rare genetic condition with a wide range of possible presentations, effective collaboration among various specialties, comprehensive diagnostic evaluations, and patient-centered follow-up are essential in navigating the complexities involved in caring for such patients. This case was presented to raise awareness among physicians regarding the ongoing challenges that may arise in managing individuals with Goldenhar syndrome. It serves as a reminder that personalized and holistic care should be provided to patients with congenital anomalies and complex medical histories, alongside other genetic conditions.
